# A comparison study on feature selection of DNA structural properties for promoter prediction

**DOI:** 10.1186/1471-2105-13-4

**Published:** 2012-01-07

**Authors:** Yanglan Gan, Jihong Guan, Shuigeng Zhou

**Affiliations:** 1 Department of Computer Science and Technology, Tongji University, Shanghai, China; 2 Shanghai Key Lab of Intelligent Information Processing and School of Computer Science, Fudan University, Shanghai, China

## Abstract

**Background:**

Promoter prediction is an integrant step for understanding gene regulation and annotating genomes. Traditional promoter analysis is mainly based on sequence compositional features. Recently, many kinds of structural features have been employed in promoter prediction. However, considering the high-dimensionality and overfitting problems, it is unfeasible to utilize all available features for promoter prediction. Thus it is necessary to choose some appropriate features for the prediction task.

**Results:**

This paper conducts an extensive comparison study on feature selection of DNA structural properties for promoter prediction. Firstly, to examine whether promoters possess some special structures, we carry out a systematical comparison among the profiles of thirteen structural features on promoter and non-promoter sequences. Secondly, we investigate the correlations between these structural features and promoter sequences. Thirdly, both filter and wrapper methods are utilized to select appropriate feature subsets from thirteen different kinds of structural features for promoter prediction, and the predictive power of the selected feature subsets is evaluated. Finally, we compare the prediction performance of the feature subsets selected in this paper with nine existing promoter prediction approaches.

**Conclusions:**

Experimental results show that the structural features are differentially correlated to promoters. Specifically, DNA-bending stiffness, DNA denaturation and energy-related features are highly correlated with promoters. The predictive power for promoter sequences differentiates greatly among different structural features. Selecting the relevant features can significantly improve the accuracy of promoter prediction.

## Background

The advent of the second generation sequencing greatly speeds up the accumulation of genome data. One of the most important tasks to understand such tremendous data is to functionally annotate genomes and analyze gene regulatory networks [1], for which, one pre-requisite step is to identify promoters from genomic sequences [2,3]. Promoters refer to crucial control regions surrounding transcription start sites (TSSs), which are the basis of transcription initiation and are responsible for steering the binding of RNA polymerase [4,5]. A thorough understanding of promoter regions can provide valuable insights into how, where and when transcription takes place.

Concerning how RNA polymerase exactly locates a promoter region at the initial stage of transcription, the presumption is that promoter sequences possess some special properties distinctive from the properties of the surrounding non-promoter regions. Previous studies mainly focus on DNA sequence compositional features. These studies have found that some local sequence compositional signals are specific to core promoter regions [6], such as CpG islands (CGIs) [7], TATA boxes [8], CAAT boxes [6], some specific transcription factor binding sites (TFBSs) [9], pentamer matrix [8] and oligonucleotides [10]. Based on these features, a variety of computational methods have been proposed for promoter prediction. Although much progress has been achieved, recent studies strikingly reveal that existing promoter prediction methods have several common limitations [6,11]. In fact, due to the complexity and heterogeneity of promoter architectures, there are only a limited number of promoters that exactly match the consensus motifs. The sequence motifs are not adequate to identify promoters, which results in a high number of false-positives when handling a whole genome. The local sequence compositional signals alone can not accurately discriminate promoters from non-promoters [12]. The selection of appropriate biological signals to predict promoters remains a field of intense investigation.

Recently, the studies of DNA crystal structures have brought to light the fact that structural properties of DNA sequences play important roles in different genome functions [13,14]. The second-order structural information encoded in promoter regions could be recognized by RNA polymerase [15]. Specifically, the curvature and bendability of DNA sequences may condition a favorable or inhibitory chromatin environment for the binding of RNA polymerase, and may further contribute to the transcription process [16]. Previous studies have also shown that the eukaryotic core promoters indeed have distinct structural properties when compared with coding or non-regulatory sequences [17-20], which presents a feasibility to explore promoter sequences from a structural perspective. Up to now, researchers have analyzed different structural features, including duplex free energy [21], stacking energy [22], DNA denaturation [23], duplex disrupt energy [24], protein deformation [25], Z-DNA [26], DNA-bending stiffness [27], A-philicity [28], nuclesome position [29], propeller twist [25], protein-DNA twist [30], B-DNA twist and bendability [31]. Each of these features has been respectively used to predict promoters [18,20]. As the high dimensionality of feature space will deteriorate the accuracy and time complexity of prediction models, it is costly to combine all these features together for promoter analysis. Meanwhile, biologists are practically inclined to know which features are more correlated with promoters. However, this issue has seldom been addressed.

Thus, in this paper, we systematically compare thirteen structural features of promoters and non-promoters, and propose a feature selection framework to explore which structural features are more related to promoter sequences. By taking advantage of various feature selection techniques, a small subset of highly discriminative features are selected and further utilized to predict promoters. From the comparative analysis, we observe that promoter sequences possess some specific structural features, like DNA-bending stiffness, duplex free energy and duplex disrupt energy. The results of different filter and wrapper feature selection methods indicate that energy-related features and DNA-bending stiffness appear more frequently in the selected feature subsets, indicating that these structural features have a closer connection to promoter sequences and the features differentiate in capability for promoter prediction. This finding is in line with the classification accuracy based on each individual feature. Furthermore, the experimental results demonstrate that the selected feature subsets can significantly improve the sensitivity and accuracy of promoter prediction.

## Results and discussion

### Comparison of structural patterns of different features

**Figure 1:**
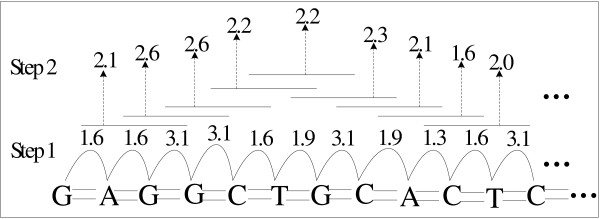
**The schematic illustration of converting a sequence into a numerical vector according to the conversion schema**. For all thirteen structural features, the conversion schemas are respectively obtained through specific physical or chemical experiments, which are summarized from different literatures [18,46].

**Figure 2:**
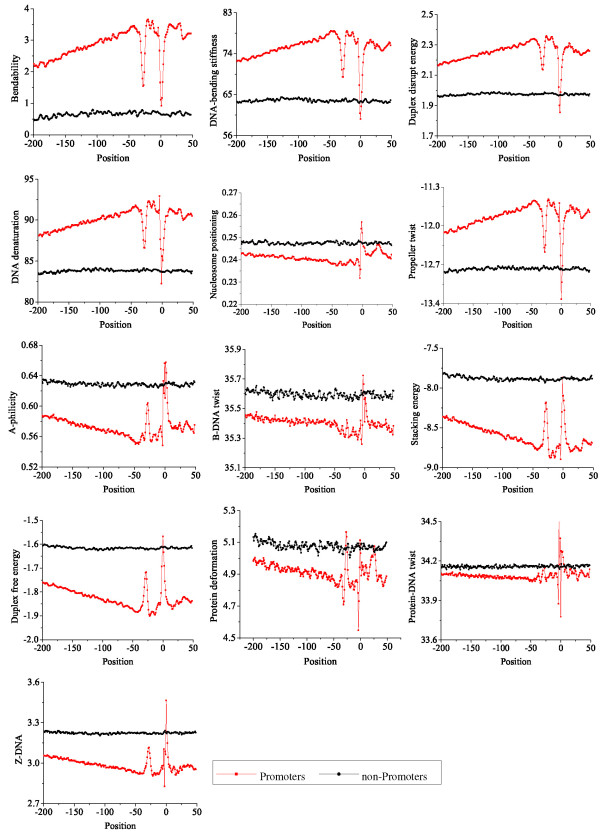
**The structural profiles of thirteen different features along the promoter and non-promoter regions**. The structural profiles are plotted according to the average value on each position. Each panel represents a structural feature, and the feature name is shown on the left side of the panel.

Different from traditional promoter prediction methods that are mostly based on sequence compositional features, in this paper we first carry out an extensive comparison between promoter and non-promoter sequences in order to explore whether promoters possess specific structures. Here, our investigation focuses on thirteen different kinds of structural features, including duplex free energy, stacking energy, DNA denaturation, duplex disrupt energy, protein deformation, Z-DNA, DNA-bending stiffness, A-philicity, nucleosome position, propeller twist, protein-DNA twist, B-DNA twist and bendability. For these features, different structure models have been derived from various biochemical experiments. According to these models, DNA sequences can be transformed into distinct structural profiles, as illustrated in Figure 1. Although these structural models are based on dinucleotides or trinucleotides, several studies have proven that these structural features are different in terms of the nucleotide information, and offer additional thermo-physical information [20,32,33]. Based on the conversion schemas, we transform the promoter and non-promoter sequences into numerical vectors and examine their similarities and differences with regard to these structural features on the DBTSS human TSS dataset (see Methods). For all these thirteen kinds of structural features, we plot and compare the structural profiles of promoters and non-promoters (Figure 2).

Through a comprehensive analysis, we obtain some interesting observations. First, for each feature, the structural values along promoter sequences are different from those along non-promoter sequences. Second, promoters have specific patterns of structural profiles, with overall stable values and two sharp troughs or peaks. The first clear trough (peak) locates at the position -30 bp upstream of TSS, where the TATA-binding protein (TBP) binds. The second trough (peak) is around the TSS. These two troughs (peaks) may guide transcription apparatus to locate the appropriate initiation site, which is in agreement with the fact that promoters bear a special structure that is essential to the assembly of the transcription machinery. On the contrary, the structural profiles of non-promoters do not exhibit such patterns. The different patterns may originate from the strongly conserved sequences, such as the TATA box around the -30 bp position and the Inr element at the TSS position. However, recent studies indicate that the presence of these elements is not enough to explain the specific structural patterns of promoters. Promoter sequences without the TATA element still have a clear trough in the structural profile of DNA denaturation, although the trough is not as deep as that with TATA box [20]. This finding suggests that promoter sequences can denature more easily than non-promoter sequences, no matter whether they contain those elements or not. In our previous study [34], the analysis on CpG-related and non-CpG related promoters shows that the two different kinds of promoter sequences exhibit similar structural patterns. As a result, we may conclude that the structural features are not only affected by the specific elements such as TATA box, Inr element and CpG island, but also by long-range combinations of nucleotides. Third, as shown in Figure 2, there are two types of structural features according to the profile patterns: either a large trough stretching over the TSS region is visible, such as the plots in the first two rows, or a peak is visible, such as the features in the last three rows. These distinctive peak or trough regions are possibly useful for identifying TSSs. Furthermore, compared with the non-promoter sequences, the structural patterns of promoters with regard to some features are distinctive and clear, such as energy-related features, DNA-bending stiffness and DNA denaturation. By analyzing the structural models, we observe that these features are closely related with the stability of promoter sequences. In contrast, the profiles of B-DNA twist, protein deformation and protein-DNA twist are ambiguous and noisy. These structural features have different capabilities of differentiating promoters and non-promoters. These findings suggest that a variety of possible mechanisms may lead to promoter recognition by RNA polymerase. The binding of RNA polymerase could result from the DNA sequence preferences. Alternatively, promoter recognition may also be guided by the boundaries of the specific structural peaks or troughs around the TSSs.

### Comparison of classification performance of different structural features

The above analysis implies that the structures of DNA sequences are important signals in promoter recognition of RNA polymerase, and integrating structural features may be helpful for promoter prediction. In order to identify the most promising features, we move forward to test how well each individual feature can differentiate promoters and non-promoters. The experiments are conducted on the DBTSS human promoter dataset. For each feature, we respectively convert the positive promoter and negative non-promoter sequences into corresponding numerical feature vectors. Because of the outstanding classification performance of the support vector machine (SVM) classifier, we apply it to discriminate promoters from non-promoters. We train thirteen SVM classifiers corresponding to all these different structural features and test their classification performance, which is evaluated by three measures, including sensitivity, specificity and F-measure.

**Figure 3:**
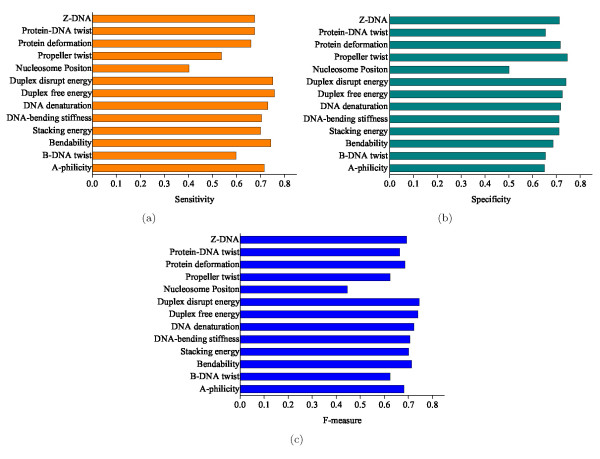
**Performance comparison of different structural features**. Sensitivity (Se), specificity (Sp) and F-measure of the classifiers built on thirteen kinds of structural features. These structural features exhibit different predictive power for promoters.

Figure 3 shows the five-fold cross-validation performance of all these thirteen structural features on the human dataset. The experimental results indicate that these structural properties are indeed different in predictive power for promoters. Generally, protein deformation, DNA-bending stiffness and protein-DNA twist have fairly high sensitivity values, but specificity values are slightly lower than those of the other features. On the contrary, duplex free energy, DNA denaturation, Z-DNA and DNA-bending stiffness strike a balance between sensitivity and specificity, leading to much higher values of F-measure.

### Ranking structural features by filter methods

To identify the discriminative structural features for promoter prediction, we adopt different filter feature selection methods to select feature subsets from the thirteen kinds of structural features. First, each sequence (length 251 bp) is converted to thirteen numerical vectors corresponding to the thirteen different structural features. Since the values of these features are at different levels, we respectively normalize these vectors into the range 0[1]. Second, we combine these feature vectors into an integrated vector whose dimension is 13*251, and add a Boolean dimension as class label with 'true' for promoters and 'false' for non-promoters. Third, various filter selection methods based on four evaluation metrics, including information gain (IG), Chi Square (CHI), ReliefF and Correlation-based Feature Selection (CFS), are used to select the most discriminative dimension subsets (see Methods). Then, all these dimensions are ranked based on the scores that are assigned by the feature selection methods. For the selected dimension subset, we calculate the ratio of each feature in the subset, that is, the ratio of the number of dimensions related to this feature over the total number of dimensions in the selected dimension subset. Only the top-ranked features are used for further classification purpose. In order to obtain a general trend, for each evaluation criterion, we select several dimension subsets with growing dimensionality, including 100, 200, 300, 400 and 500. Furthermore, we use the SVM classifier to evaluate the predictive ability of each selected subset.

Table 1 presents the distribution of each feature in the resulted dimension subsets based on different evaluation criteria. Here we show the selected dimension subsets with the dimensionality of 200 and 300. Specifically, since CFS is a multivariate evaluation metric, it is unfeasible to rank each dimension subset. Genetic search strategy is applied to search the feature space. For CFS, we choose the two selected subsets with dimensionality 227 and 319 to compare with the other three criteria. Although the selected subsets vary from case to case, a general trend is observed. There is a marked enrichment of DNA bending stiffness, duplex free energy and duplex disrupt energy in the selected dimension subsets. Whereas, some features such as B-DNA twist and protein-DNA twist seldom appear in the selected dimension subsets. In the case of information gain and Chi Square, B-DNA twist is not selected in the subsets, whereas some structural and thermodynamic features related to energy content and stiffness are more specific for promoters. These results indicate that the structural features are not equally correlated with promoter sequences. Promoter sequences are characterized not only by sequence features, but also by high order chromatin structures. For example, as GC base pairing is stronger than AT base pairing, GC content of DNA sequences is related with the stability-related features, such as DNA bending-stiffness, DNA-denaturation and duplex free energy. Since regions with high free energy are more unstable than those with low thermodynamic energy, the highly unstable peaks surrounded by the overall stable regions are hard to bend for nucleosome formation, and easy to denature. These specific structural features are important indications for the binding of RNA polymerase.

**Table 1:** The feature selection results of the filter methods

	IG		CHI		ReliefF		CFS	
	
Structural features	200	300	200	300	200	300	200	300
A-philicity	2	5	2	5	5	4.7	7.9	8.8
B-DNA twist	0	0	0	0	2	2.7	6.2	4.7
Bendability	2	3	2	3	6	6.7	8.4	7.8
Stacking energy	3	3	3	3	7	7.7	5.7	9.4
DNA-bending stiffness	19.5	17.3	20	17.3	13	12	17.6	9.4
DNA denaturation	11	14.7	10.5	14.7	9	9.7	18.9	10.1
Duplex free energy	21.5	20	22	20	15	15.7	11	8.5
Duplex disrupt energy	23.5	17.7	23	17.7	17.5	16.3	5.7	5
Nucleosome position	2	2.3	2	2.3	3	4	2.6	7.5
Propeller twist	6	8	6	7.7	5.5	5.3	5.3	9.1
Protein deformation	4	4	4	4	6.5	5.7	3.1	7.5
Protein-DNA twist	2	1.3	2	1.3	3.5	4	4.4	4.7
Z-DNA	3.5	3.7	3.5	4	7	5.7	3.1	7.5

The distribution of each feature in the dimension subset selected by different filter methods. The percentage of each feature in a selected subset is calculated as follows: (the number of dimensions related to the feature/the total number of dimensions in the selected dimension subset)*100%.

As filter selection methods rank features totally based on the inherent characteristics of datasets, we need to further evaluate whether the selected features are effective for classification. Therefore, the selected feature subsets are further used to build SVM classifiers. Table 2 shows the performance of the SVM classifiers of each selected feature subset. Each evaluation criterion includes two subsets with dimensionality 200 and 300. In the case of information gain, it gains the best performance when the top 400 dimensions are selected. While for ReliefF and CHI, the subset with 300 dimensions is preferable. As for CFS criterion, subsets with dimensionality 319 or 274 are selected, and the subset with 319 dimensions is better for classification.

**Table 2:** The performance evaluation of the feature subsets selected by different filter feature selection methods

	IG		CHI		ReliefF		CFS	
	
Peformance	200	300	200	300	200	300	200	300
Specificity	0.759	0.759	0.755	0.76	0.751	0.772	0.733	0.746
Sensitivity	0.704	0.718	0.707	0.704	0.718	0.743	0.727	0.731
F-measure	0.73	0.738	0.73	0.731	0.734	0.757	0.73	0.738
ROC score	0.74	0.745	0.738	0.741	0.74	0.762	0.731	0.741

Based on the feature subsets selected by four filter feature selection methods, we respectively build SVM classifiers and compare the performance of these classifiers.

### Ranking structural features by wrapper methods

Wrapper methods provide alternatives to perform multivariate feature selection, which are dependent on the target classifiers whose accuracies are utilized to evaluate the candidate feature subsets. Thus, the selected feature subsets are closely related to specific classifiers, and the classification performance is usually better than that of filter methods. As it is prohibitively costly to evaluate all possible subsets, we adopt the genetic search strategy to search for the optimal subsets, in combination with SVM and kNN classifiers respectively.

**Figure 4:**
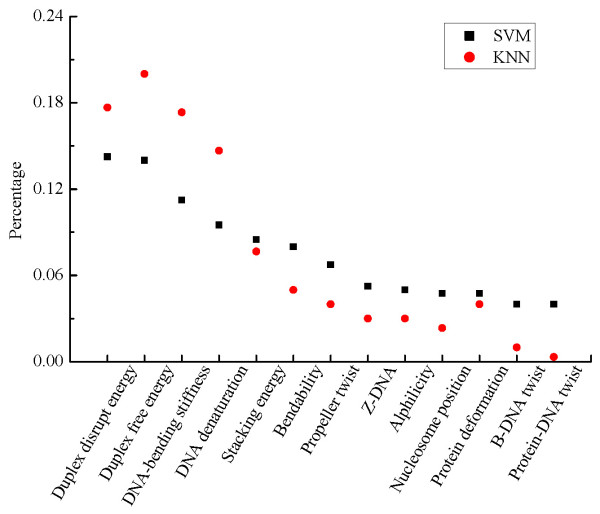
**The feature selection results of the wrapper methods**. The distribution of each feature in the selected feature subsets by the wrapper feature selection methods. Two different target classifiers, SVM and kNN are used in the wrapper feature selection.

Table 3 summarizes the performance of the wrapper feature selection methods. The first two rows report the classification results with the wrapper feature selection, while the third and fourth rows are the classification results without feature selection. From the table, we observe that the classifiers with the wrapper feature selection achieve much better classification performance than those without feature selection. Compared with the results of the filter feature selection methods, the wrapper methods are better in most cases, except ReliefF. For the wrapper methods, we also compute the ratio of each feature in the selected subset as mentioned above. From the distribution plots in Figure 4, the subsets selected by the wrapper methods indicate the similar trend as the filter methods. The ratios of duplex free energy, DNA-bending stiffness and DNA denaturation in the selected subsets are much higher than those of the other features, indicating that they are more predictive for promoter prediction.

**Table 3:** The performance evaluation of the wrapper feature selection methods

Classifier	Wrapper	Specificity	Sensitivity	F-measure	ROC score	Dimensionality
SVM	SVM	0.761	0.723	0.742	0.786	320
KNN	KNN	0.705	0.731	0.718	0.745	300
SVM	NULL	0.733	0.72	0.731	0.732	All
KNN	NULL	0.637	0.753	0.69	0.723	All

The performance comparison of two classifiers (SVM and kNN) for promoter classification: with the wrapper feature selection (the first two rows) vs. without wrapper feature selection (the last two rows).

### Classification performance comparison with other existing methods

In this section, we evaluate the effectiveness of the selected structural feature subsets in promoter prediction (termed as FSPP in this paper), and compare them with nine existing promoter prediction methods. We select five classical methods that perform well in previous comparative studies [12,35], including FirstEF [36], Eponine [37], DragonGSF [7], McPromoter [8] and ARTS [38]. These methods are all traditional, in the sense that they were mainly built on sequence compositional features, such as CpG island, TATA box and a number of motifs. FirstEF is based on quadratic discriminant analysis of promoters, the first exon and the first donor site. Eponine constructs a relevance vector machine based on TATA box in a G+C rich domain. DragonGSF utilizes artificial neural network. McPromoter also adopts neural network to predict promoters, but uses combined features from different segments. By using support vector machine, ARTS integrates several compositional features and two structural features. Meanwhile, we include four newly proposed methods that depend on structural features to predict promoters. EP3 directly uses a deviation from the average structural value to locate the promoter regions [20]. Observing the distinct structural patterns of promoter regions, PNNP develops a pattern-based promoter prediction method [34]. Based on DNA melting temperature, Profisi predicts promoters using simple thresholding [39]. ProSOM applies self-organizing maps to distinguish promoters from non-pomoters [40].

To achieve an unbiased evaluation of different promoter prediction methods, we conduct the performance comparison on the CAGE dataset, which is different from our training dataset. All the predictions are subject to the same evaluation criterion. If a prediction is within 500 bp of an annotated TSS, we call it a true positive hit. All existing prediction methods are tested with their default settings, which are provided as the optimal parameters by their developers. The empirical performance results of different promoter prediction methods are shown in Table 4. Though McPromoter has a considerable specificity, its sensitivity is low, which leads to a bad F-measure score. On the contrary, FirstEF achieves a balanced sensitivity and specificity. From Table 4, we observe that the promoter prediction methods based on structural features perform better than those methods utilizing sequence compositional features. However, as these methods use only one structural feature to predict promoters, the improvement of performance is not significant. By adopting the feature selection methods to select the most discriminative feature subsets from all these structural features we studied, the sensitivity and specificity can be greatly improved.

**Table 4:** Performance comparison of promoter prediction methods on the human genome

Methods	Specificity	Sensitivity	F-measure
FirstEF	0.415	0.448	0.431
DragonGSF	0.686	0.357	0.470
McPromoter	0.623	0.204	0.307
EP3	0.565	0.413	0.477
Profisi	0.604	0.392	0.475
ARTS	0.672	0.381	0.486
Eponine	0.671	0.367	0.475
ProSom	0.573	0.414	0.481
PNNP	0.593	0.433	0.501
FSPP(ReliefF-300)	0.657	0.528	0.585
FSPP(Wrapper-SVM)	0.662	0.536	0.592

These methods include nine existing promoter prediction methods and our prediction methods based on the filter and wrapper feature selection.

## Conclusions

Since most current promoter prediction methods rely on local sequence compositional signals, they suffer from low accuracy in identifying promoters. This paper systematically analyzes promoter sequences from various structural perspectives. We investigate thirteen different structural features to examine whether promoter sequences exhibit some specific structures for promoter recognition by RNA polymerase. By converting the promoter and non-promoter sequences into numerical vectors, the structural profiles of different features are explicitly presented. These structural profiles manifest some useful findings. On one hand, compared with non-promoter regions, promoter regions indeed show different structural values and patterns. We infer that structural properties may be effective to differentiate promoters from non-promoters. On the other hand, the profile patterns of all structural features are not equally distinct for promoters and non-promoters. The profiles of some features such as duplex free energy, duplex disrupt energy and DNA-bending stiffness are much more distinct than those of the other features.

Comparative classification analysis based on individual feature further suggests that the predictive power of these structural features are quite different. In order to quantify the difference, we turn to various feature selection methods including four typical filter methods and two wrapper methods based on SVM and kNN. As filter methods are independent on the classifiers, they are more general for use. For the wrapper methods, the feature selection results depend on the classifiers and the prediction performance may vary. Overall, B-DNA twist and protein-DNA twist seldom appear in the resulted feature subsets. Whereas the ranks of DNA-bending stiffness and energy-related features based on these feature selection methods are higher than those of the other features, indicating a strong correlation with promoters. This result is consistent with our further correlation analysis among all these structural features. The pairwise Pearson correlation coefficients indicate that the energy related features, including Duplex free energy, Duplex disrupt energy and DNA denaturation, are highly correlated with each other (Additional File 1, Table S1). Also, the DNA-bending stiffness and bendability are closely related to the energy-related features. As a result, these features show similar presence in the selected feature subsets. By analyzing these thirteen kinds of features in a uniform feature selection framework, the results show that the energy-related features and DNA-bending stiffness are highly correlated with promoter sequences. These specific structures of promoters can be taken as clues for promoter prediction. Furthermore, rather than use a single structural feature, we use the selected feature subsets to predict promoters. The performance of promoter prediction confirms that the selected feature subsets are informative in promoter prediction.

The findings perhaps not only have biological meaning but also facilitate the applications of more powerful promoter prediction models on various genomes.

## Methods

### Core promoter datasets

To extensively analyze the structural features of promoter sequences, we retrieve a collection of promoter sequences from DBTSS (version 7.0) [41]. DBTSS is built on experimentally validated TSSs, which is mainly based on full-length cDNA transcripts, and the 5'-ends of oligo-cap selected cDNAs are experimentally determined [42]. This dataset provides the best training data to recent computational studies in genomic annotation and promoter analysis. DBTSS includes the promoter sequences of several species. Here, our analysis is based on one of the largest dataset, the human dataset. We extract the sequences from 200 bp upstream to 50 bp downstream flanking TSSs as the promoter sequences. Through filtering, we obtain 11,682 experimentally validated human TSSs. Correspondingly, we retrieve the same number of non-promoter sequences. In order to avoid the position bias in extracting sequences from the human genome, we build a randomized non-promoter dataset by shuffling the real promoter sequences. From the promoter dataset, we obtain a first-order Markov model that preserves the dinucleotide frequencies. Furthermore, based on the Markov model, the randomized non-promoter sequences are generated.

To unbiasedly compare the performance of different promoter prediction methods, the comparative evaluation is conducted on another dataset - the CAGE dataset, which is different from our training dataset and has a wider coverage of human genome. The CAGE dataset is based on the cap analysis gene expression (CAGE) technique and is retrieved from the Riken institute website [3]. As previous study, only tag clusters identified by two or more tags are included in our analysis [35]. Mapping these tags to human genome allows us to obtain 181,046 unique human TSSs. The whole genome (hg19) is retrieved via the UCSC Genome Browser [43].

### Calculating structural profile

Because of important roles in different key biological processes, many studies have focused on biophysical understanding of the intrinsic structural properties of DNA sequences. Recent experimental analyses have shown that, in some manner, eukaryotic core promoters are indeed marked by specific structures comparing with coding or non-regulatory sequences. For instance, the duplex free energy content at TSS positions is much higher than that at the other positions. As a region with high free energy is more active than a region with low thermodynamic energy content [21], the stable regions may provide a contrasting background for the highly unstable peaks, and make the peaks prominent for guiding transcription apparatus to locate the appropriate transcription sites [34]. Also, the propeller twist capacity measures the rigidity of a sequence [44]. A region with high negative propeller twist is rigid, which makes this region hard to wrap around a nucleosome and facilitates RNA polymerase to bind in this area [45]. Besides these two features, we also investigate other eleven structural properties, including stacking energy [26], DNA denaturation [22], duplex disrupt energy [24], protein deformation [25], Z-DNA [23], bending stiffness [27], A-philicity [28], nucleosome position [29], protein-DNA twist [25], B-DNA twist [30], and bendability [31].

In order to quantitatively analyze structural properties of promoters, we convert the retrieved promoter and non-promoter sequences into numerical structural profiles corresponding to different structural features. For each feature, the calculation of structural profile is divided into two steps, as illustrated in Figure 1. We first transform each DNA sequence into a numerical vector. Each dinucleotide (or trinucleotide) is replaced by its corresponding structural value [18,20]. The transformation models for different structural features are obtained from various bio-chemical experiments, and are summarized as the validated conversion schemas by Florquin et al. [18]. Specifically, since genome-wide nucleosome maps for different species have been recently published, we use the new in vivo human nucleosome positioning data from Schones et al.'s study to calculate the structural profiles related to nucleosome positioning [46]. We take all the nucleosome-bound sequences from their collection, and then align these sequences with regard to their centers. By using the same method as in the previous study [47], we obtain the new nucleosome parameters. Accordingly, the new structural profiles related to nucleosome positioning are calculated. Second, we use a sliding window approach to smooth the raw profiles, with a window-size of 3 nt and a step-size of 1 bp. When the window slides along a sequence, a vector of structural values is output. We plot the average value on each position to get the structural profile of the sequence with regard to each feature. At last, every sequence has thirteen structural profiles corresponding to all these structural features.

### Feature selection framework

Feature selection has been an important issue in machine learning, data mining and statistics fields, and has drawn much attention in diverse bioinformatics applications [48]. Here, to gain a deeper insight into the structural properties of promoters, to avoid the overfitting problem and to improve the performance of promoter prediction models, we adopt different feature selection techniques to analyze these structural features we studied. We identify features that are highly correlated with promoter sequences and effective in promoter recognition. According to the combination mode between feature selection and classification process, feature selection techniques generally fall into the following two categories: wrapper and filter methods [49].

A wrapper method depends on a target classifier to identify the optimal feature subset that provides the target classifier with the best classification performance. The feature selection process consists of a search in the feature space guided by the performance of the target classifier. While bringing good accuracy for the final classifier, wrapper methods are computationally expensive. Filter methods are independent of any target classifier. These methods select features by looking only at the intrinsic properties of the data, such as class separability or correlation between features and classes. First, a feature relevance score is calculated, and the low-scoring features are filtered. Afterwards, selected features can be presented as input to a classification algorithm. Hence, the filter feature selection methods are computationally efficient and can easily scale to high-dimensional datasets.

**Figure 5:**
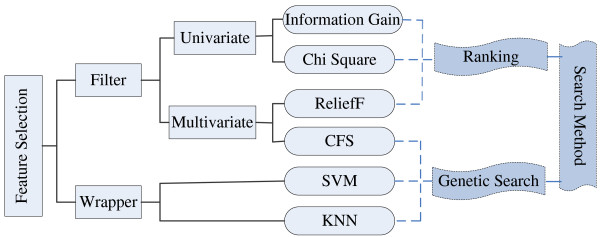
**The framework of the feature selection process**. The feature selection methods are used in our analysis to choose the most related structural features of promoters. According to the relation with the target classifier, these methods are classified into two categories, filter and wrapper methods. Each method is composed of two critical elements: evaluation criteria of features and search strategy in the feature space.

Generally, feature selection techniques differ from each other with respect to two main aspects: evaluation criterion of features and search strategy in the feature space. To gain a systematical and unbiased analysis, here we take advantage of various feature selection techniques based on different evaluation functions in conjunction with search strategies, as shown in Figure 5. For filter methods, four different evaluation criteria are adopted to evaluate features, assigning a score to each feature that suggests how valuable the feature is for classification. The four evaluation criteria include two univariate criteria: information gain (IG) and Chi Square (CHI), and two multivariate criteria: ReliefF and Correlation-based Feature Selection (CFS) [48]. Then, all features are ranked by their scores and the highly relevant features are obtained according to a given threshold. At last, these features subsets selected by different filter methods are evaluated by classification performance. As the calculation of correlation coefficients among features is costly and it is prohibitively expensive to rank each feature subset, we adopt the genetic search strategy for CFS, which is different from the other criteria. For wrapper models, the target classifier and search strategy are critical elements. We respectively use SVM and kNN as target classifiers, utilizing the accuracy of classification to measure the quality of selected features. Meanwhile, the genetic search algorithm is applied to search for optimal feature subsets in the feature space. This combination is due to the following reasons. In literature, SVM classifier is widely used because of its high classification accuracy [50], and the instance-based classifier kNN is quite efficient [51]. Genetic search is effective in high-dimensional search space. All our feature selection methods are developed based on an open-source data-mining tool, Weka [52]. Finally, we report the 5-fold cross validation estimate of classification accuracy.

### Performance measures

Sensitivity (*Se*) and specificity (*Sp*) are two criteria widely used to evaluate the performance of promoter prediction models [12]. Additionally, we utilize F-measure and ROC score to measure the overall performance of the prediction models. These measures are defined as below:

(1) $$Se=\frac{TP}{TP+FN}$$

(2) $$Sp=\frac{TP}{TP+FP}$$

(3) $$F-measure=\frac{2\left(Se\cdot Sp\right)}{Se+Sp}$$

*TP*, *FP *and *FN *represent the numbers of true positives, false positives and false negatives respectively. Generally, sensitivity is the proportion of correct predictions of TSSs over all experimental TSSs. Specificity is the proportion of correct prediction of TSSs out of all counted positive predictions. The higher the value of *Se *is, the more false positives may be reported, and the lower the value of *Sp *is. It is a trade-off to balance sensitivity and specificity. F-measure is a single measure that can compare prediction methods with different sensitivity and specificity. Meanwhile, the quality of a classifier can be evaluated by ROC score, which computes the area under the ROC curve [53]. The value of the ROC score ranges from zero to one, with a score of 0.5 corresponding to random guess and a score of 1.0 indicating perfect separation. The ROC score indicates the accuracy with which a classifier separates promoters from non-promoters.

## Authors' contributions

YLG conceived of the study, performed the experiments, analyzed the data and drafted the manuscript. JHG participated in the design of the study and analyzed the data. SGZ analyzed the data and helped draft the manuscript. All authors read and approved the manuscript.

## Supplementary Material

Additional file 1 **Table S1**. Pairwise Pearson correlation coefficients among structural profiles of thirteen different structural features across the human genome. Click here for file
