# Supplementary material for: A comparison study on feature selection of DNA structural properties for promoter prediction
Source: BMC Bioinformatics. 2012 Jan 7;13:4. doi: 10.1186/1471-2105-13-4 (PMC3280155; doi:10.1186/1471-2105-13-4)
Supplement: Additional file 1 — Table S1. Pairwise Pearson correlation coefficients among structural profiles of thirteen different structural features across the human genome. [file 1471-2105-13-4-S1.PDF]

	DNA denaturation	Propeller twist	DNA-bending stiffness	Duplex disrupt energy	Bendability	Z-DNA	Stacking energy	Duplex free energy	Aphilicity	B-DNA twist	Protein deformation	Protein-DNA twist	Nucleosome position
DNA denaturation	1												
Propeller twist	0.753	1											
DNA-bending stiffness	0.842	0.749	1										
Duplex disrupt energy	0.817	0.641	0.805	1									
Bendability	0.306	0.599	0.212	-0.023	1								
Z-DNA	-0.778	-0.778	-0.769	-0.759	-0.275	1							
Stacking energy	-0.839	-0.744	-0.803	-0.797	-0.372	0.756	1						
Duplex free energy	-0.857	-0.73	-0.862	-0.825	-0.213	0.781	0.857	1					
A-philicity	-0.713	-0.714	-0.727	-0.534	-0.436	0.719	0.63	0.666	1				
B-DNA twist	-0.387	-0.121	-0.262	-0.268	0.07	0.279	0.198	0.249	0.574	1			
Protein deformation	0.012	-0.122	0.021	0.071	-0.312	-0.045	0.012	-0.031	0.145	-0.033	1		
Protein-DNA twist	-0.347	-0.666	-0.416	-0.173	-0.627	0.336	0.258	0.288	0.562	0.199	0.307	1	
Nucleosome position	0.742	0.753	0.702	0.574	0.627	-0.758	-0.733	-0.716	-0.681	-0.52	-0.024	-0.173	1
